# Identification of Synthetic Urine by Analysis of Stable Carbon and Nitrogen Isotope Ratios and Comparison to Established GC‐MS/MS and LC‐MS/MS Analysis

**DOI:** 10.1002/dta.70106

**Published:** 2026-06-18

**Authors:** Frank Hülsemann, Laura Franke, Dirk K. Wissenbach, Gregor Fußhöller, Mario Thevis

**Affiliations:** ^1^ Institute of Biochemistry German Sport University Cologne Cologne Germany; ^2^ Jena University Hospital, Institute for Forensic Medicine Friedrich Schiller University Jena Jena Germany

## Abstract

Manipulation of urine samples is a recurring issue in doping control and forensic analyses, for instance through the use of synthetic urine products designed to mimic human urine. This study evaluated analytical approaches for identifying synthetic urine and mixtures of synthetic and authentic urine: gas chromatography tandem mass spectrometry (GC‐MS/MS) for analysis of endogenous urinary steroids, elemental analyzer isotope ratio mass spectrometry (EA‐IRMS) of carbon and nitrogen, and urea‐nitrogen compared to an established liquid chromatography mass spectrometry (LC‐MS/MS) method incorporating synthetic urine markers. All methods correctly identified synthetic urine in a double‐blind sample set. As an initial testing procedure in doping control, GC‐MS/MS identified synthetic urine samples through absence of endogenous steroids. However, EA‐IRMS was superior to MS/MS methods in identifying mixtures of synthetic and authentic urine. Synthetic urine isotope ratios of total urinary carbon and nitrogen and/or urea nitrogen (δ^13^C ≤ −29.7‰, δ^15^N ≤ +0.4‰) were clearly differentiated from authentic urine isotope ratios (δ^13^C ≥ −26.1‰, δ^15^N ≥ +1.6‰), reflecting the synthetic origin of constituents. Mixtures of synthetic and authentic urine up to 50:50 displayed isotope signatures inconsistent with human urine, enabling reliable detection of adulteration. The LC‐MS/MS approach detected mixed samples with high proportions of synthetic urine by combining biomolecule profiling with synthetic urine‐specific markers. Overall, the findings demonstrate that EA‐IRMS is a complementary tool for identifying synthetic or manipulated urine samples, especially when traditional biomarkers or steroid profiles are inconclusive. The method enhances the reliability of doping control and forensic urine authenticity testing.

## Graphical abstract

This study compared GC‐MS/MS, EA‐IRMS, and LC‐MS/MS for detecting synthetic and manipulated urine samples in doping control and forensic analysis. EA‐IRMS proved particularly effective for identifying mixtures of synthetic and authentic urine, providing a reliable complement to established authenticity testing methods.

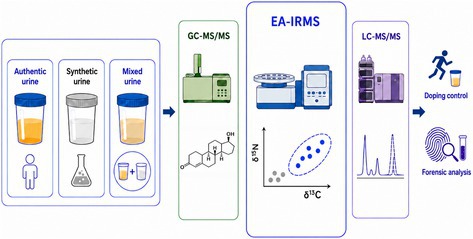

## 1 Introduction

In both doping control and forensic analysis, identifying manipulated urine samples is a common problem. Although in doping control, urine sampling is performed under observed collection, there are rare cases when altered samples are received by anti‐doping laboratories [1]. Manipulation can include swapping samples, adding substances, or substitution with liquids, such as water, apple juice, alcohol‐free beer, or synthetic products [1, 2, 3, 4, 5, 6]. The substitution and/or adulteration of doping control samples by an athlete or another person constitutes a violation of the World Anti‐Doping Agency (WADA) anti‐doping rules and is listed under class “M2.1 Physical and chemical manipulation” on the “Prohibited list” [7]. Anti‐doping laboratories are encouraged to uncover attempts of sample manipulation, including adulteration and sample swapping [1, 8, 9].

Over the past few years, synthetic urine (SU) products have become available. These products are used to manipulate (substitute) urine samples in drug abuse testing and potentially in sports for doping tests. SUs imitate the color, pH, and specific gravity of human urine to a certain extent and contain biomolecules such as creatinine and urea [5, 10, 11]. Indicator for analytical detections of SUs are the absence of biomolecules (indirect SU markers) that are usually present in human urine such as carnitines and steroids [1, 2, 11, 12, 13, 14] and/or the presence of substances that are usually not found in human urine but are present in synthetic products (direct SU markers), such as benzisothiazolinone, triethylene glycol, tetraethylene glycol [11], as well as polypropylene glycols (PPG) [12], and PPG+16, the SU marker 255 (SUM255), and carbendazim [12, 15]. In doping control analysis, absence or very low concentrations of urinary endogenous steroids are a strong indicator of urine sample manipulation by substitution or dilution of the sample. However, absence or low concentrations of endogenous steroids in a doping control sample do not prove sample manipulation per se. Additional analytical methods are necessary for definitive verification of a sample manipulation. Several methods supporting the detection of SUs have recently been developed and published. In a forensic context, these methods use rapid screening techniques, such as temperature control, shaking, urine dip sticks, and automated assays, as well as more comprehensive analytical methods, such as microscopy and LC‐MS/MS [5, 6, 11, 12, 13, 14, 15, 16, 17, 18]. Dip sticks and automated SU screening methods are beneficial in terms of effort, cost, and analysis time; however, they are presumptive and may produce false‐positive or ‐negative results [5, 12, 15, 18]. Thus, confirmation procedures are needed for these approaches, e.g., using MS‐based methods [5, 11, 15, 18]. Previously, an LC‐MS/MS method that included both indirect and direct SU markers was introduced, expanded upon, and tested in different studies using double‐blind and authentic urine (AU) specimens [12, 13, 15]. This method was found to be reliable, providing 100% sensitivity and specificity for SU identification [12]. Nevertheless, to maintain the effectiveness of this method, the SU product market must be constantly monitored for new products, and specific direct SU markers must be identified to prove SU usage. In forensic science and doping control analysis, isotope ratio mass spectrometry (IRMS) is used to distinguish between natural and synthetic origins of biomolecules and can therefore help to identify SU without the need for direct markers [19, 20]. The first aim of this study was to compare the results of stable isotope analysis of SU and authentic urinary samples to the aforementioned LC‐MS/MS method for SU identification as a reference method. This comparison used the same double‐blind specimen set as in [11, 12]. The second aim was to compare the established steroid GC‐MS/MS analysis used in doping control to the LC‐MS/MS method, again using the same sample set. The third aim was to analyze mixtures of different ratios of SU and AU using all three methods to assess their ability to identify such mixtures and their limitations.

## 2 Materials and Methods

### 2.1 Chemicals

Acetonitrile (ACN), formic acid, and water (all LC–MS‐grade) were acquired from Fisher Chemical (Schwerte, Germany); ammonium formate was purchased from Sigma‐Aldrich (Steinheim, Germany). Eight SU products were purchased for a previous study [12] and reused in this study: Urin King, red point (SU1), Urin King, black point (SU2), and Urin King, blue point (SU3) (all Maxharaj & Saeb GbR, Munich, Germany); “Synthetischer Urin ‐ DIN EN 1616/DIN EN ISO 20696” (SU4) and “Synthetischer Human Urin (Original Imitat) urinfarben” (SU5) (both SU e.K., Eberdingen‐Nußdorf, Germany); Artificial Urine, Pickering (SU6) (LCTech GmbH, Obertaufkirchen, Germany); CleanUrin, green point (SU7), and CleanUrin, yellow point (SU8) (both CleanU, Eberdingen‐Nußdorf, Germany). The “DRI Creatinine‐Detect Probenvaliditätstest” was obtained from Thermo Fisher Scientific (Darmstadt, Germany).

Xanthydrol was obtained from Sigma‐Aldrich, Steinheim, Germany; glacial acetic acid (Merck, Darmstadt, Germany) and methanol (VWR, Darmstadt, Germany) were analytical grade.

### 2.2 Urine Specimens

This study used 51 specimens from a previous study, consisting of 43 urine specimens (AU) from volunteers (30× female, 13× male) and eight SU products from the Austrian/German market [12]. These 51 specimens were used to generate two specimen sets: For specimen set A, the 43 AUs and eight SUs were randomly intermixed and blinded, and afterwards analyzed by GC‐MS/MS and EA‐IRMS. Set B consisted of mixtures of the eight SUs, respectively, with the pooled 43 urine specimens in three different ratios: 10% SU with 90% urine, 50% SU with 50% urine, and 90% SU with 10% urine. In contrast to set A, for set B, proportions of the mixtures as well as corresponding SU products were known prior to EA‐IRMS, GC‐MS/MS, LC‐MS/MS, and creatinine (CREA) analysis. Volunteers who provided the urine specimens consented to (follow‐up) analysis. All specimens were thawed twice (due to the analysis in the previous study), otherwise kept frozen at −20°C before analysis. The stability of the analyzed endogenous biomolecules (phenylalanine, tryptophan, propionylcarnitine, butyrylcarnitine, isovalerylcarnitine, hexanonylcarnitine, phenylacetylglutamine, heptanoylcarnitine, octanoylcarnitine, and indolacetylglutamine) was checked with LC‐MS/MS following the two freeze–thaw cycles and compared to previous study results [12, 21]. In consensus with previous results, nine out of 10 analytes were found stable, while indolacetylglutamine had decreased levels.

### 2.3 Creatinine Analysis

Creatinine (CREA; Jaffe reaction, linearity of the test: 7.8–4200 mg/L obtained from the user manual) was analyzed in sample set B for completeness on an AU480 auto‐analyzer (Beckman Coulter, Krefeld, Germany).

### 2.4 EA‐IRMS

For stable isotope analysis of total carbon and nitrogen, 8 to 20 μL of a sample was transferred into a tin capsule and dried under reduced pressure over phosphorous pentoxide. Additionally, for each sample, urea was precipitated using the xanthydrol method, described elsewhere [22]. The measurements were carried out using elemental analysis isotope ratio mass spectrometry (EA‐IRMS). The elemental analyzer (Eurovektor EA 3000, Hekatech, Wegberg, Germany) was equipped with a Zero Blank Revolver Autosampler (Blisotec, Jülich, Germany) and connected to a Delta V isotope‐ratio mass spectrometer via a ConFlo IV interface (both Thermo Scientific, Bremen, Germany). The working gas for carbon (CO_2_, purity 4.5, Linde, Pullach, Germany) was calibrated with IAEA‐CH‐6 (−10.449‰), IAEA‐CH‐7 (−32.151‰), and IAEA 600 (−27.771‰), all from the International Atomic Energy Agency (IAEA, Vienna, Austria). The δ^13^C values are expressed in per mil (‰) relative to Vienna Pee Dee Belemnite (VPDB). Nitrogen isotope ratios are expressed relative to atmospheric nitrogen (AIR). Working standard gas for nitrogen (N_2_, purity 5.0 from Linde, Munich, Germany) was scale calibrated using IAEA‐N‐1 (+0.4‰) and IAEA‐N‐2 (+20.3‰) for δ^15^N values (both from IAEA, Vienna, Austria). During the analyses, the performance of the instrument was regularly checked with blanks and an internal working standard (creatine monohydrate, Alzchem, Trostberg, Germany). The standard deviations of repeated measurements of the working standard were ±0.1‰ for carbon and ±0.2‰ for nitrogen.

### 2.5 GC‐MS/MS

To determine the concentration of endogenous urinary steroids, samples from specimen sets A and B were prepared according to established doping control procedures. In brief, the screening protocol included an enzymatic hydrolysis step with β‐glucuronidase followed by liquid–liquid extraction (LLE) and evaporation. Subsequently, trimethylsilylation was performed followed by GC‐MS/MS analysis [23]. Samples were analyzed for the markers of the steroid profile: androsterone (A), etiocholanolone (Etio), 5α‐androstane‐3α,17β‐diol (5αAdiol), 5β‐androstane‐3α,17β‐diol (5βAdiol), testosterone (T), and epitestosterone (E) as well as the endogenous reference compounds (ERCs) pregnanediol (PD) and 11β‐hydroxy‐androsterone (11‐OH‐A) and additionally dehydroepiandrosterone (DHEA).

### 2.6 LC‐MS/MS

An established LC‐MS/MS method [13], targeting 10 endogenous biomolecules (phenylalanine, tryptophan, propionylcarnitine, butyrylcarnitine, isovalerylcarnitine, hexanonylcarnitine, phenylacetylglutamine, heptanoylcarnitine, octanoylcarnitine, and indolacetylglutamine) as well as direct SU markers (polypropylene glycols [PPG], PPG+16, SUM255, and carbendazim [15]) was previously performed for specimen set A, and details are given in [12] with the corresponding results as well as in Table S1. For specimen set B, LC‐MS/MS analysis was now performed with the same method, which served as a reference method for comparison to GC‐MS/MS and EA‐IRMS analysis. LC‐MS/MS data analysis was performed in Xcalibur (version 4.0.2.). A specimen was evaluated as authentic by LC‐MS/MS, if ≥ 6 endogenous biomolecules and no SU markers were detected.

### 2.7 Statistical Analyses

Statistical analyses were performed in R (version 4.3.0, R Core Team, 2023). Individual comparisons were conducted between two independent groups using the Mann–Whitney *U* test (Wilcoxon rank‐sum test). Statistical significance was defined as *p* < 0.001.

## 3 Results and Discussion

### 3.1 GC‐MS/MS of Endogenous Steroids in Double‐Blind Specimens

Like in routine doping control analysis, all 51 specimens of set A were analyzed for endogenous steroids usually present in human urine including the markers of the urinary steroid profile [9]. Eight samples (# 08, 13, 24, 29, 30, 38, 47, and 51) out of the 51 samples of set A were suspicious and clearly distinguishable from the other (human urine) samples as they contained no detectable amounts of endogenous steroids (see Table S2). Thus, all 51 set A specimens were classified correctly as AU or suspicious samples in comparison to LC‐MS/MS results from the previous study (see Table S1) [12].

In specimen set B, all analyzed samples contained detectable amounts of urinary steroids with increasing concentrations of all steroids while decreasing amounts of SU in the mixtures (Table S3). In routine doping control analysis, none of these samples would have been classified as “suspicious” due to the presence of endogenous steroids in each individual sample.

### 3.2 Total Carbon and Nitrogen Isotope Ratios in Double‐Blind Specimens

**FIGURE 1:**
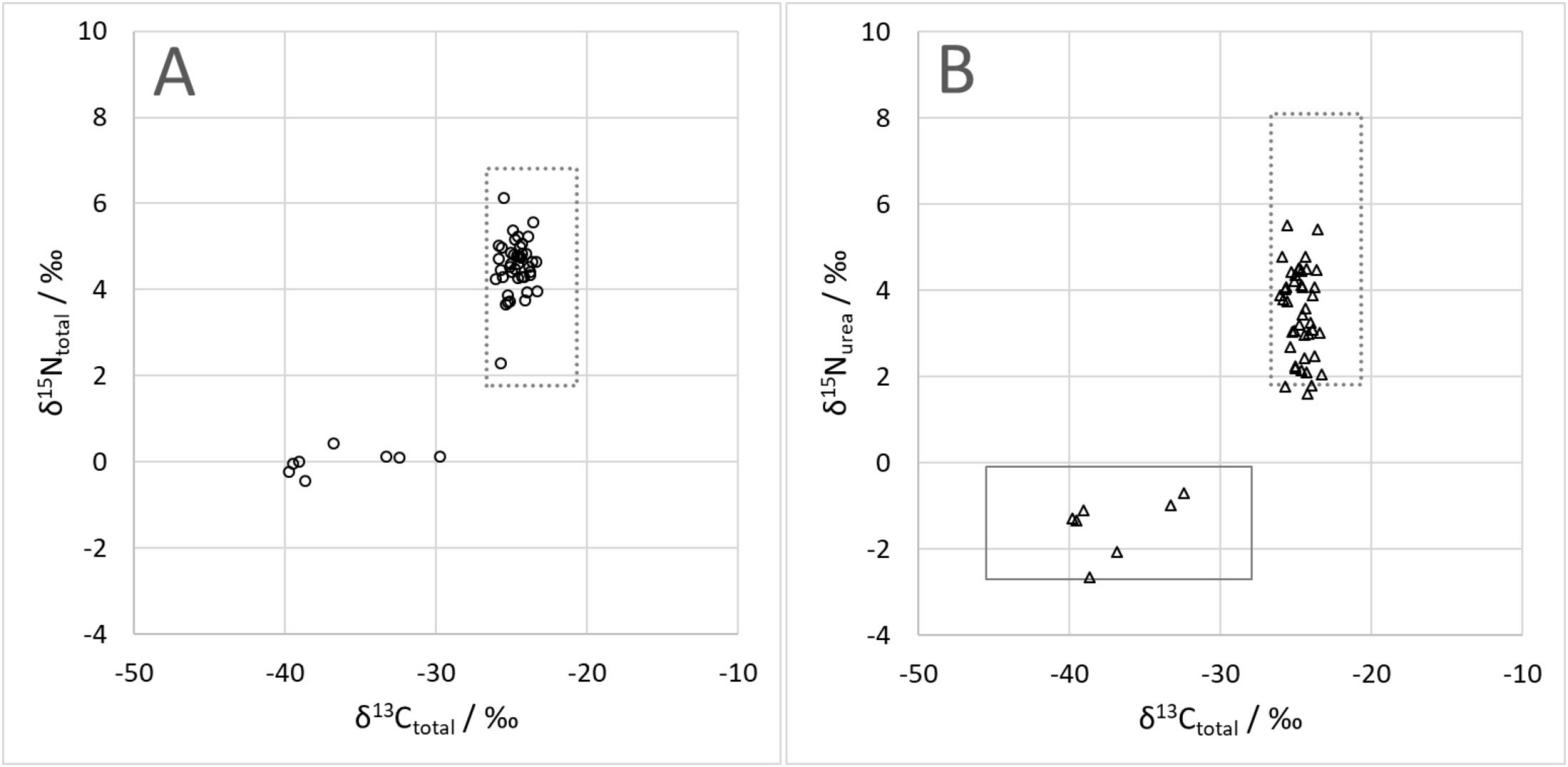
Isotope ratios of samples from specimen set A (blinded study) for total carbon vs. total nitrogen (A, circles) and total carbon vs. urea nitrogen (B, triangles). Shown rectangles display reference areas for human urine (dashed line) and synthetic urea (solid line).

All 51 samples of set A contained sufficient amounts of carbon and nitrogen for stable isotope analysis. Regarding total carbon (δ^13^C_total_) and nitrogen isotope ratios (δ^15^N_total_) for set A, two distinct subgroups were identified (Figure 1; for δ‐values, see Table S2). The major subgroup containing 43 samples showed δ^13^C_total_ values between −26.05‰ and −23.31‰ and δ^15^N_total_ values between +2.29‰ and +6.13‰. These carbon and nitrogen isotope ratios are in the typical range for urine samples of contemporary European/US humans [24, 25, 26, 27]: reported total urine isotopic values for contemporary humans range from −26.6‰ (vegan diet, Europe [26]) to −20.7‰ (C4‐plant influenced diet in the United States, [26]) for carbon and +1.8% (vegan diet, Europe [26]) to +8.3‰ (fish as major protein source, Europe [25]) for nitrogen (Table 1).

**TABLE 1:** Reported δ^13^C and δ^15^N values of human total urine from literature as reference values, *n* is the number of subjects.

	δ^13^C_total_/‰	δ^15^N_total_/‰	Study	*n*	Country
Petzke et al. 2009 [23]	−24.3 ± 0.6 to −23.8 ± 0.4^a^	+4.5 ± 0.5 to 4.5 ± 0.5^a^	Diet intervention	14	Germany
Kuhnle et al. 2012 [24]	−25.0 to −22.9	+3.4 to +6.8	Diet intervention	45	United Kingdom
Dierkes et al. 2023 [25]	−26.64 to −21.06	+1.76 to +5.76	Diet survey	72	Germany
O'Brien et al. 2025 [26]	−21.9 to −20.7	+3.6 to +4.4	Controlled feeding study	113	United States

^a^ Mean values ± standard deviation.

The minor subgroup consisting of eight set A samples (# 08, 13, 24, 29, 30, 38, 47, and 51) showed δ^13^C_total_ values between −39.77‰ and −29.73‰ and δ^15^N_total_ values between −0.43‰ and +0.44‰ (Table S2). Such carbon and nitrogen isotope ratios are not consistent with human isotopic signatures but are found for products of synthetic origin. The δ^13^C values below −30‰ point towards a fossil origin of carbon as it is found for synthetic products like polymers [28], urea, or creatine‐monohydrate [29]. All 51 set A specimens were assigned correctly to be AU or SU when compared to the LC‐MS/MS method from the previous study (see Table S1) [12]. The two groups revealed highly statistical differences as well as for carbon and for nitrogen isotope ratios (both *p* < 0.001).

**FIGURE 2:**
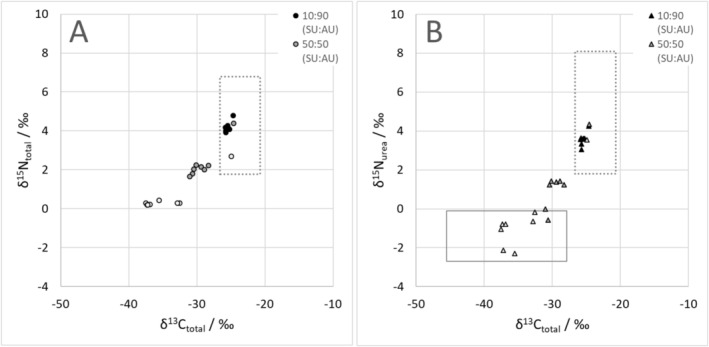
Isotope ratios of specimen set B (mixtures of synthetic [SU] and authentic urine [AU] samples) for total carbon vs. total nitrogen (A, circles) and total carbon vs. urea nitrogen (B, triangles). Shown rectangles display reference areas for human urine (dashed line) and synthetic urea (solid line).

For specimen set B, those mixtures with 90% SU and 10% urine showed δ^13^C_total_ values between −37.52‰ and −24.91‰ and δ^15^N_total_ values between +0.20‰ and +2.70‰ (Table S3). Except for one 90:10 mixture, all of these samples were allocated to the minor subgroup of set A, containing the SU samples (Figure 2). These samples were statistically not differentiable compared to the SU samples of set A for carbon (*p* = 0.61) and nitrogen (*p* = 0.01). Compared to the AU samples, all these mixtures revealed highly statistical differences, both for carbon and nitrogen isotope ratios (both *p* < 0.001).

For the samples with 90% AU and only 10% SU, statistical analyses indicated no significant difference for carbon (*p* = 0.001) as well as for nitrogen compared to AU samples (*p* = 0.006), although minor differences between these groups concerning mean values and variances exist. If the carbon and nitrogen isotope ratios of the 90:10 (AU:SU) mixtures are compared individually with the AU samples of set A, no difference could be detected. All of these mixtures revealed highly statistic differences for carbon and nitrogen compared to the SU samples (both *p* < 0.001).

All but one mixture containing equal volumes of AU and SU showed δ^13^C_total_ and δ^15^N_total_ values not usually observed when testing human urine samples and were statistically different for carbon and nitrogen compared to the AU samples (both *p* < 0.001). Statistical analyses revealed no significant difference between the 50:50 (SU:AU) mixtures for carbon (*p* = 0.004), but for nitrogen (*p* < 0.001) compared to SU samples.

One set of mixtures (product #6) showed more or less identical δ^13^C_total_ and δ^15^N_total_ values for all three mixtures, located in the AU subgroup. This is due to the absence or very low concentrations of organic molecules in this SU product, as no urea could be detected by the xanthydrol method (Table S2), and no creatinine was detectable by automated analysis (Table S1) in this sample. These mixtures were not incorporated in the statistical analyses.

### 3.3 Urea Nitrogen Isotope Ratios in Double‐Blind Specimens

All 51 set A specimens were additionally analyzed using the xanthydrol method [22] to obtain the isotopic composition of the urea nitrogen (δ^15^N_urea_). For two samples (# 37 and 38), EA‐IRMS analysis of urea nitrogen was impossible due to insufficient precipitation of dixanthylurea (see Table S2). The other samples showed comparable, albeit slightly lower, nitrogen isotope ratios for urea as had been determined for total urine. Forty‐two samples yielded δ^15^N_urea_ values between +1.59‰ and +5.50‰. Such values are typical for urinary urea of German inhabitants, which have been determined in our laboratory to range between +1.4‰ to +8.2‰ (unpublished data). In contrast to these values, seven samples (# 08, 13, 24, 29, 30, 47, and 51) showed δ^15^N_urea_ values below 0.0‰, clearly differentiable from human urinary urea. These samples showed isotope ratios like synthetic urea available as certified working standard for EA‐IRMS, which range from −48.63‰ to −27.9‰ for carbon and from −2.91‰ to −0.07‰ for nitrogen (Table 2). Regarding δ^15^N_urea_, the difference for the two groups of samples was highly statistically significant (*p* < 0.001).

**TABLE 2:** Measured or certified (IVA Analysentechnik, Meerbusch, Germany) δ^13^C and δ^15^N values for synthetic urea (EA‐IRMS working standard) as reference. Given are the certificate or batch number, whichever is known.

δ^13^C/‰	δ^15^N/‰	Supplier/certificate/batch
−27.9^a^	−0.3^a^	IVA/‐/158535
−45.5^a^	−1.5^a^	Sigma/‐/092K0170
−43.3	−0.6	IVA/290365 and 258279/‐/
−43.99	−0.82	IVA/420891/‐/
−39.20	−0.07	IVA/453524/‐/
−36.54	−2.35	IVA/332489/‐/
−35.46	−2.38	IVA/346530/‐/
−42.18	−0.69	IVA/391235/‐/
−36.59	−2.2	IVA/318862/‐/
−45.38	−0.85	IVA/‐/3137
−37.02	−2.91	IVA/291488/‐/
−41.30	−0.32	IVA/236531/‐/
−37.32	−0.45	IVA/214009/‐/
−48.63	−0.30	IVA 180170/‐/
−39.73	−0.73	IVA/147273/‐/
−40.81	−0.49	IVA/128785/‐/

^a^ Own measurements per EA‐IRMS.

For specimen set B, a similar pattern like for total urine nitrogen was observed: The 90:10 (SU:AU) mixtures showed δ^15^N_urea_ values below 0.0‰, except for the mixture of product #6, which contained very low amounts of urea. For this product, the precipitated urea originates mostly from the AU. Statistical analyses for the other 90:10 (SU:AU) mixtures revealed no statistical difference compared to the SU samples (*p* = 0.32), but highly statistic difference compared to the authentic human urine samples (*p* < 0.001). The 50:50 mixtures showed a trend towards the AU samples. Statistical analyses revealed highly significant differences to both, synthetic and AU samples (both *p* < 0.001). The 10:90 (SU:AU) mixtures revealed no statistically significant difference compared to the AU samples (*p* = 0.83), but were highly statistically different from the SU samples (*p* < 0.001).

### 3.4 LC‐MS/MS of Human Urine and SU Mixtures

With respect to the LC‐MS/MS method, all mixtures with 90% SU and 10% AU were found suspicious as they all contained less than six endogenous biomolecules (see Table S4). For those mixtures containing SU1, SU2, SU3, SU7, and SU8, additionally, SU markers were detected, thus being classified as diluted with SU. In mixtures containing SU1, SU2 or SU3 the so far unidentified direct SU marker SUM255 was detected. In mixtures containing SU7 and SU8, PPG were detected, a direct SU marker class, which might be contained in SU products because of its viscous, colorless, odorless, and solvent features [30]. For the mixtures with 50% SU and 50% AU, at least six endogenous biomolecules were detected, while for four of them (containing SU1, SU2, SU7, and SU8), also SU markers were detected, thus being classified as mixed AU and SU specimens. The other four 50:50 SU mixtures (containing SU3, SU4, SU5, and SU6) were assigned to be unsuspicious AU, because no direct SU markers were detected in these samples. This highlights the necessity of identifying direct SU markers in all available SU products when applying the LC‐MS/MS method to reveal SU:AU mixtures. Specimens containing 10% SU and 90% AU were found to contain more than six EBs and, with the exception of those containing SU7 and SU8, no direct SU markers were observed. Thus, mixtures with SU1–SU6 were evaluated as AU, and SU7 and SU8 as AU potentially diluted with SU. For completeness, CREA measurement was performed in all mixtures. CREA values were ≥ 200 mg/L, as given by national and international guidelines [31, 32], for all mixtures except the 90% SU6 and 10% AU mixture with CREA = 90 mg/L (see Table S4).

## 4 Conclusions

All three methods were able to distinguish between pure synthetic and AU samples: GC‐MS/MS of urinary steroids due to their absence in the SU samples, as has been shown before for LC‐MS/MS endogenous biomolecules and additional detection of direct SU markers [12] and EA‐IRMS of carbon and nitrogen isotope ratios due to the synthetic isotopic signature of the SU samples' main constituents. Mixtures of synthetic and AU could not be detected by GC‐MS/MS of urinary steroids, whereas LC‐MS/MS was able to identify SU content in some mixtures, depending on the different concentrations of SU markers. By EA‐IRMS, mixtures up to 50% synthetic and 50% authentic human urine could be identified as suspicious samples due to their unusual total carbon and nitrogen isotopic compositions. As previously demonstrated, SU products contain varying amounts of creatinine, urea [12], and sometimes uric acid [5] to imitate human urine composition. The carbon and nitrogen isotope ratios of the SU samples in total and for urea nitrogen indicate a synthetic origin of both carbon (fossil) and nitrogen (air), respectively. Thus, SU products and even mixtures up to 50% SU can be differentiated from pure authentic human urine samples using EA‐IRMS. Analyzing suspicious urine samples in doping control or forensic applications by EA‐IRMS can help to identify the origin and potential source of samples provided as authentic human urine samples as a complementary method.

## Conflicts of Interest

The authors declare no conflicts of interest.

## Supporting information




**Table S1:** Randomization and de‐blinding of specimen set A. Given are the sample‐ID and the results of creatinine (CREA) and LC‐MS/MS measurement (EB = endogenous biomolecules, SU = synthetic urine) from the previous study [12] as well as de‐blinding of SU and AU specimens.

**Table S2:** Specific gravity (SG), concentrations of endogenous steroids (androsterone [A], etiocholanolone [Etio], 5α‐androstane‐3α,17β‐diol [5αAdiol], 5β‐androstane‐3α,17β‐diol [5βAdiol], testosterone [T], epitestosterone [E], pregnanediol [PD], 11β‐hydroxy‐androsterone [11‐OH‐A], and dehydroepiandrosterone [DHEA]) and δ^15^N and δ^13^C results for specimen set A. Samples printed in bold are suspicious due to the absence of detectable amounts of steroids and isotope ratios.

**Table S3:** Specific gravity (SG), concentrations of endogenous steroids (androsterone [A], etiocholanolone [Etio], 5α‐androstane‐3α,17β‐diol [5αAdiol], 5β‐androstane‐3α,17β‐diol [5βAdiol], testosterone [T], epitestosterone [E], pregnanediol [PD], 11β‐hydroxy‐androsterone [11‐OH‐A], and dehydroepiandrosterone [DHEA]) and δ^15^N and δ^13^C results for specimen set B. SU = synthetic urine; AU = authentic urine, SU:AU = percentage of mixtures.

**Table S4:** Evaluation of specimen set B by LC‐MS/MS. Specimen set B consisted of mixtures of synthetic urine (SU) and authentic urine (AU) in the rations 90:10, 50:50, and 10:90. Given are measured creatinine values (CREA), number of endogenous biomolecule (EB) detections, and detected direct SU markers.

## Data Availability

The data that support the findings of this study are available in the Supporting Information of this article.

## References

[1] T. Piper , H. Geyer , F. Huelsemann , et al., “When Is a Sample a Urine Sample? Markers for Urine Sample Authenticity Assessment in Sports Drug Testing,” Bioanalysis 17 (2025): 1355–1363, 10.1080/17576180.2025.2580273. 41200853 PMC12694900

[2] M. Thevis , H. Geyer , G. Sigmund , and W. Schänzer , “Sports Drug Testing: Analytical Aspects of Selected Cases of Suspected, Purported, and Proven Urine Manipulation,” Journal of Pharmaceutical and Biomedical Analysis 57 (2012): 26–32, 10.1016/j.jpba.2011.09.002. 21955645

[3] M. Thevis , O. Krug , H. Geyer , K. Walpurgis , N. Baume , and A. Thomas , “Analytical Challenges in Sports Drug Testing,” Analytical and Bioanalytical Chemistry 410 (2018): 2275–2281, 10.1007/s00216-018-0934-9. 29445832

[4] T. Piper , H. Geyer , N. Haenelt , F. Huelsemann , W. Schaenzer , and M. Thevis , “Current Insights Into the Steroidal Module of the Athlete Biological Passport,” International Journal of Sports Medicine 42 (2021): 863–878, 10.1055/a-1481-8683. 34049412 PMC8445669

[5] S. Vikingsson , S. T. Krauss , R. E. Winecker , R. R. Flegel , and E. D. Hayes , “Update on Urine Adulterants and Synthetic Urine Samples to Subvert Urine Drug Testing,” Journal of Analytical Toxicology 46 (2022): 697–704, 10.1093/jat/bkac029. 35639619

[6] D. K. Wissenbach and A. E. Steuer , “Advances in Testing for Sample Manipulation in Clinical and Forensic Toxicology—Part A: Urine Samples,” Analytical and Bioanalytical Chemistry 415 (2023): 5101–5115, 10.1007/s00216-023-04711-w. 37145190 PMC10404192

[7] World Anti‐Doping Agency , Prohibited List (World Anti‐Doping Agency, 2024), https://www.wada‐ama.org/sites/default/files/2024‐09/2025list_en_final_clean_12_september_2024.pdf.

[8] World Anti‐Doping Agency , International Standard for Laboratories (World Anti‐Doping Agency, 2021), https://www.wada‐ama.org/sites/default/files/resources/files/isl_2021.pdf.

[9] World Anti‐Doping Agency , Technical Document—TD2021EAAS (World Anti‐Doping Agency, 2021), https://www.wada‐ama.org/sites/default/files/2022‐01/td2021eaas_final_eng_v_2.0.pdf.

[10] M. Pfäffli , S. König , and S. Srivastava , “Synthetischer Urin,” Rechtsmedizin 26 (2016): 103–108, 10.1007/s00194-015-0076-8.

[11] M. M. Goggin , C. M. Tann , A. Miller , A. Nguyen , and G. C. Janis , “Catching Fakes: New Markers of Urine Sample Validity and Invalidity,” Journal of Analytical Toxicology 41 (2017): 121–126, 10.1093/jat/bkw119. 27881620

[12] L. Franke , C. Fuczik , M. Hubig , F. T. Peters , and D. K. Wissenbach , “Evaluation of Biochemical Assays and Optimization of LC–MS‐MS Analysis for the Detection of Synthetic Urine,” Journal of Analytical Toxicology 48, no. 1 (2024): 37–43, 10.1093/jat/bkad082. 37933588

[13] J. Kluge , L. Rentzsch , D. Remane , F. T. Peters , and D. K. Wissenbach , “Systematic Investigations of Novel Validity Parameters in Urine Drug Testing and Prevalence of Urine Adulteration in a Two‐Year Cohort,” Drug Testing and Analysis 10 (2018): 1536–1542, 10.1002/dta.2447. 29956490

[14] P. B. Kyle and J. Kaur , “Evaluating Novel Markers for Specimen Validity Testing,” Archives of Pathology and Laboratory Medicine 144 (2020): 168–171, 10.5858/arpa.2019-0197-OA. 31755779

[15] L. Franke , C. Fuczik , and D. K. Wissenbach , “Evaluation of Diagnostic Sensitivity and Specificity of a New Automatized Assay for Indirect Synthetic Urine Identification,” Drug Testing and Analysis 17 (2025): 2022–2031, 10.1002/dta.3908. 40405346

[16] A. Mina , L. McNeice , M. Rennie , S. Banukumar , and S. Vazquez , “How to Screen for Adulterated and Synthetic Urine Samples When Testing for Drugs of Abuse? A Study to Evaluate and Integrate Axiom Assay in Specimen Validity Testing Protocol,” EC Pharmacology and Toxicology 10 (2022): 33–40.

[17] F. Silva , I. Kaileh , and G. A. Hobbs , “A New Automated Assay for the Detection of Synthetic Urine in Drug Testing,” Drug Testing and Analysis 11 (2019): 926–930, 10.1002/dta.2596. 30920168

[18] V. J. Kim , C. K. Okano , C. R. Osborne , D. M. Frank , C. T. Meana , and M. S. Castaneto , “Can Synthetic Urine Replace Authentic Urine to “Beat” Workplace Drug Testing?” Drug Testing and Analysis 11 (2019): 331–335, 10.1002/dta.2497. 30194711

[19] W. Meier‐Augenstein , Stable Isotope Forensics: Methods and Forensic Applications of Stable Isotope Analysis (John Wiley and Sons, 2017), 10.1002/9781119080190.

[20] A. T. Cawley and U. Flenker , “The Application of Carbon Isotope Ratio Mass Spectrometry to Doping Control,” Journal of Mass Spectrometry 43 (2008): 854–864, 10.1002/jms.1437. 18523972

[21] L. Franke , F. T. Peters , and D. K. Wissenbach , “Long Time Stability of 35 Small Endogenous Biomolecules in Dried Urine Spotted on Various Surfaces and Environmental Conditions,” Forensic Science International 339 (2022): 111420. 35985138 10.1016/j.forsciint.2022.111420

[22] F. Hülsemann , K. Koehler , U. Flenker , and W. Schänzer , “Do We Excrete What We Eat? Analysis of Stable Nitrogen Isotope Ratios of Human Urinary Urea,” Rapid Communications in Mass Spectrometry 31 (2017): 1221–1227, 10.1002/rcm.7891. 28466567

[23] U. Mareck , H. Geyer , G. Opfermann , M. Thevis , and W. Schänzer , “Factors Influencing the Steroid Profile in Doping Control Analysis,” Journal of Mass Spectrometry 43 (2008): 877–891, 10.1002/jms.1457. 18570179

[24] K. J. Petzke and S. Lemke , “Hair Protein and Amino Acid ^13^C and ^15^N Abundances Take More Than 4 Weeks to Clearly Prove Influences of Animal Protein Intake in Young Women With a Habitual Daily Protein Consumption of More Than 1 g per kg Body Weight,” Rapid Communications in Mass Spectrometry 23 (2009): 2411–2420, 10.1002/rcm.4025. 19603474

[25] G. G. Kuhnle , A. M. Joosen , C. J. Kneale , and T. C. O'Connell , “Carbon and Nitrogen Isotopic Ratios of Urine and Faeces as Novel Nutritional Biomarkers of Meat and Fish Intake,” European Journal of Nutrition 52 (2013): 389–395, 10.1007/s00394-012-0328-2. 22406837 PMC3549402

[26] J. Dierkes , S. Dietrich , K. Abraham , et al., “Stable Isotope Ratios of Nitrogen and Carbon as Biomarkers of a Vegan Diet,” European Journal of Nutrition 62 (2023): 433–441, 10.1007/s00394-022-02992-y. 36087137 PMC9899720

[27] D. M. O'Brien , L. S. Freedman , P. Rivera , et al., “Urine Stable Isotope Ratios Are Associated With Proportional Intakes of Animal Protein and Added Sugars in a 15‐D Controlled Feeding Study,” Journal of Nutrition 155 (2025): 3949–3956, 10.1016/j.tjnut.2025.08.013. 40812475 PMC12443068

[28] D. Berto , F. Rampazzo , C. Gion , et al., “Preliminary Study to Characterize Plastic Polymers Using Elemental Analyser/Isotope Ratio Mass Spectrometry (EA/IRMS),” Chemosphere 176 (2017): 47–56, 10.1016/j.chemosphere.2017.02.090. 28254714

[29] F. Hülsemann , U. Flenker , M. Parr , H. Geyer , and W. Schänzer , “Authenticity Control and Identification of Origin of Synthetic Creatine‐Monohydrate by Isotope Ratio Mass Spectrometry,” Food Chemistry 125 (2011): 767–772, 10.1016/j.foodchem.2010.09.018.

[30] K. Bischoff , “Propylene Glycol,” in Small Animal Toxicology, eds. M. E. Peterson and P. A. Talcott (Elsevier, 2013), 763–767.

[31] W. Schubert , V. Dittmann , and J. Brenner‐Hartmann , Urteilsbildung in der Fahreignungsbegutachtung: Beurteilungskriterien, 3rd ed. (Kirschbaum‐Verlag, 2013).

[32] Department of Health and Human Services , 42 CFR Chapter I, Mandatory Guidelines for Federal Workplace Drug Testing Programs (Department of Health and Human Services, 2022), https://www.govinfo.gov/content/pkg/FR‐2022‐04‐07/pdf/FR‐2022‐04‐07.pdf.

