# Supplementary material for: Identification of Synthetic Urine by Analysis of Stable Carbon and Nitrogen Isotope Ratios and Comparison to Established GC‐MS/MS and LC‐MS/MS Analysis
Source: Drug Test Anal. 2026 Jun 18;18(8):1145–51. doi: 10.1002/dta.70106 (PMC13432729; doi:10.1002/dta.70106)
Supplement: Supplementary file 1 — Table S1: Randomization and de‐blinding of specimen set A. Given are the sample‐ID and the results of creatinine (CREA) and LC‐MS/MS measurement (EB = endogenous biomolecules, SU = synthetic urine) from the previous study [12] as well as de‐blinding of SU and AU specimens. [file DTA-18-1145-s001.docx]

Table S1: Randomization and de-blinding of specimen set A. Given are the sample-ID and the results of creatinine (CREA) and LC-MS/MS measurement (EB = endogenous biomolecules, SU = synthetic urine) from the previous study [12] as well as de-blinding of SU and AU specimens.

| **Sample** | **Sample-ID**  **PMID: 37933588** | **CREA** | **LC-MS/MS** | | **De-Blinding** |
| --- | --- | --- | --- | --- | --- |
|  |  | **mg/L** | **EB** | **SU Marker** |  |
| 01 | A5 | 240 | 10 |  | AU |
| 02 | A7 | 390 | 8 |  | AU |
| 03 | A19 | 270 | 9 |  | AU |
| 04 | A37 | 90 | 9 |  | AU |
| 05 | A3 | 2910 | 10 |  | AU |
| 06 | A27 | 490 | 10 |  | AU |
| 07 | A9 | 650 | 10 |  | AU |
| 08 | A24 | 230 | 0 |  | SU2 |
| 09 | A50 | 2440 | 9 |  | AU |
| 10 | A30 | 1240 | 9 |  | AU |
| 11 | A31 | 310 | 9 |  | AU |
| 12 | A12 | 410 | 9 |  | AU |
| 13 | A25 | 1100 | 0 |  | SU4 |
| 14 | A22 | 740 | 9 |  | AU |
| 15 | A16 | 1280 | 10 |  | AU |
| 16 | A18 | 270 | 9 |  | AU |
| 17 | A11 | 80 | 9 |  | AU |
| 18 | A47 | 200 | 10 |  | AU |
| 19 | A36 | 650 | 9 |  | AU |
| 20 | A20 | 130 | 9 |  | AU |
| 21 | A34 | 1230 | 10 |  | AU |
| 22 | A29 | 690 | 10 |  | AU |
| 23 | A14 | 190 | 10 |  | AU |
| 24 | A38 | 480 | 0 | Polypropylene glycols, (PPG+16) | SU7 |
| 25 | A43 | 830 | 10 |  | AU |
| 26 | A8 | 2160 | 8 |  | AU |
| 27 | A32 | 260 | 8 |  | AU |
| 28 | A42 | 980 | 10 |  | AU |
| 29 | A35 | 630 | 0 | PPG, (PPG+16) | SU8 |
| 30 | A10 | 430 | 0 |  | SU1 |
| 31 | A15 | 90 | 9 |  | AU |
| 32 | A17 | 1910 | 10 |  | AU |
| 33 | A28 | 1670 | 10 |  | AU |
| 34 | A39 | 1080 | 10 |  | AU |
| 35 | A46 | 590 | 10 |  | AU |
| 36 | A2 | 180 | 10 |  | AU |
| 37 | A45 | 80 | 9 |  | AU |
| 38 | A51 | 0 | 0 |  | SU6 |
| 39 | A40 | 110 | 10 |  | AU |
| 40 | A13 | 40 | 8 |  | AU |
| 41 | A6 | 1140 | 8 |  | AU |
| 42 | A33 | 750 | 10 |  | AU |
| 43 | A44 | 710 | 9 |  | AU |
| 44 | A23 | 160 | 9 |  | AU |
| 45 | A1 | 1630 | 9 |  | AU |
| 46 | A49 | 2180 | 10 |  | AU |
| 47 | A26 | 300 | 0 |  | SU3 |
| 48 | A48 | 270 | 9 |  | AU |
| 49 | A41 | 840 | 10 |  | AU |
| 50 | A21 | 240 | 10 |  | AU |
| 51 | A4 | 750 | 0 |  | SU5 |
