# Supplementary material for: Identification of Synthetic Urine by Analysis of Stable Carbon and Nitrogen Isotope Ratios and Comparison to Established GC‐MS/MS and LC‐MS/MS Analysis
Source: Drug Test Anal. 2026 Jun 18;18(8):1145–51. doi: 10.1002/dta.70106 (PMC13432729; doi:10.1002/dta.70106)
Supplement: Supplementary file 2 — Table S2: Specific gravity (SG), concentrations of endogenous steroids (androsterone [A], etiocholanolone [Etio], 5α‐androstane‐3α,17β‐diol [5αAdiol], 5β‐androstane‐3α,17β‐diol [5βAdiol], testosterone [T], epitestosterone [E], pregnanediol [PD], 11β‐hydroxy‐androsterone [11‐OH‐A], and dehydroepiandrosterone [DHEA]) and δ15N and δ13C results for specimen set A. Samples printed in bold are suspicious due to the absence of detectable amounts of steroids and isotope ratios. [file DTA-18-1145-s003.docx]

Table S2: Specific gravity (SG), concentrations of endogenous steroids (androsterone (A), etiocholanolone (Etio), 5α-androstane-3α,17β-diol (5αAdiol), 5β-androstane-3α,17β-diol (5βAdiol), testosterone (T), epitestosterone (E), pregnanediol (PD), 11β-hydroxy-androsterone (11-OH-A) and dehydroepiandrosterone (DHEA)) and δ^15^N and δ^13^C results for specimen set A. Samples printed in bold are suspicious due to the absence of detectable amounts of steroids and isotope ratios.

| **Sample** | **SG** | **A** | **Etio** | **5α**  **Adiol** | **5β**  **Adiol** | **T** | **E** | **PD** | **11-OH-A** | **DHEA** | **δ^15^N_total_** | **δ^15^N_urea_** | **δ^13^C_total_** |
| --- | --- | --- | --- | --- | --- | --- | --- | --- | --- | --- | --- | --- | --- |
|  |  | **ng/ml** | | | | | | | | | **‰** | | |
| 01 | 1.006 | 1454 | 820 | 24 | 83 | 15 | 9 | 163 | 549 | 11 | 5.23 | 4.44 | -24.60 |
| 02 | 1.011 | 1755 | 774 | 12 | 9 | 1 | 6 | 867 | 663 | 23 | 4.33 | 4.07 | -23.75 |
| 03 | 1.010 | 899 | 650 | 14 | 20 | 2 | 3 | 71 | 276 | 7 | 5.56 | 5.40 | -23.55 |
| 04 | 1.003 | 148 | 59 | 2 | 2 | **< LOD** | **< LOD** | 13 | 68 | **< LOD** | 3.75 | 2.98 | -24.15 |
| 05 | 1.029 | 5506 | 4877 | 85 | 69 | 9 | 63 | 793 | 3143 | 70 | 4.85 | 2.24 | -25.03 |
| 06 | 1.011 | 1186 | 1512 | 36 | 241 | 6 | 5 | 325 | 1013 | 21 | 4.75 | 4.77 | -24.36 |
| 07 | 1.014 | 541 | 925 | 4 | 7 | **< LOD** | 1 | 201 | 803 | 7 | 4.53 | 3.09 | -23.88 |
| **08** | **1.012** | **< LOD** | **< LOD** | **< LOD** | **< LOD** | **< LOD** | **< LOD** | **< LOD** | **< LOD** | **< LOD** | **-0.05** | **-1.34** | **-39.50** |
| 09 | 1.028 | 3616 | 4540 | 111 | 673 | 44 | 27 | 521 | 1849 | 65 | 4.40 | 2.47 | -23.76 |
| 10 | 1.018 | 3588 | 1957 | 97 | 94 | 14 | 38 | 211 | 874 | 44 | 5.23 | 3.88 | -23.91 |
| 11 | 1.007 | 459 | 483 | 4 | 8 | **< LOD** | 2 | 99 | 206 | 10 | 5.16 | 4.52 | -24.76 |
| 12 | 1.010 | 1429 | 622 | 18 | 14 | 3 | 5 | 52 | 1144 | 19 | 2.29 | 1.77 | -25.73 |
| **13** | **1.020** | **< LOD** | **< LOD** | **< LOD** | **< LOD** | **< LOD** | **< LOD** | **< LOD** | **< LOD** | **< LOD** | **0.44** | **-2.08** | **-36.82** |
| 14 | 1.015 | 432 | 916 | 6 | 34 | 2 | 2 | 375 | 582 | 9 | 4.64 | 4.47 | -23.62 |
| 15 | 1.023 | 4712 | 4895 | 66 | 388 | 9 | 8 | 808 | 1223 | 174 | 3.95 | 1.79 | -23.96 |
| 16 | 1.010 | 317 | 261 | 4 | 29 | 1 | 0 | 62 | 402 | 4 | 4.27 | 4.12 | -24.60 |
| 17 | 1.004 | 89 | 136 | 2 | 8 | **< LOD** | **< LOD** | 44 | 162 | **< LOD** | 6.13 | 5.50 | -25.55 |
| 18 | 1.007 | 501 | 870 | 6 | 40 | 2 | 1 | 139 | 216 | 10 | 4.28 | 3.74 | -25.59 |
| 19 | 1.011 | 202 | 590 | 3 | 31 | 2 | 1 | 52 | 330 | 5 | 4.61 | 4.36 | -25.06 |
| 20 | 1.007 | 431 | 655 | 5 | 40 | 2 | 1 | 110 | 308 | 7 | 3.73 | 3.07 | -25.11 |
| 21 | 1.021 | 644 | 1959 | 9 | 81 | 2 | 9 | 783 | 383 | 30 | 3.96 | 2.05 | -23.31 |
| 22 | 1.016 | 1473 | 1240 | 23 | 28 | 3 | 5 | 148 | 673 | 21 | 4.65 | 3.01 | -23.40 |
| 23 | 1.009 | 898 | 596 | 23 | 25 | 13 | 19 | 170 | 465 | 17 | 5.02 | 4.78 | -25.89 |
| **24** | **1.015** | **< LOD** | **< LOD** | **< LOD** | **< LOD** | **< LOD** | **< LOD** | **< LOD** | **< LOD** | **< LOD** | **0.11** | **-0.70** | **-32.43** |
| 25 | 1.019 | 324 | 332 | 5 | 13 | 1 | 1 | 70 | 680 | 6 | 4.78 | 3.43 | -24.58 |
| 26 | 1.026 | 2877 | 2854 | 101 | 121 | 36 | 29 | 513 | 2491 | 56 | 4.79 | 2.14 | -24.64 |
| 27 | 1.009 | 166 | 185 | 2 | 2 | 1 | 0 | 54 | 307 | 3 | 4.81 | 4.50 | -24.83 |
| 28 | 1.018 | 1132 | 2164 | 24 | 245 | 22 | 23 | 211 | 635 | 23 | 4.77 | 2.97 | -24.44 |
| **29** | **1.015** | **< LOD** | **< LOD** | **< LOD** | **< LOD** | **< LOD** | **< LOD** | **< LOD** | **< LOD** | **< LOD** | **0.11** | **-0.98** | **-33.30** |
| **30** | **1.015** | **< LOD** | **< LOD** | **< LOD** | **< LOD** | **< LOD** | **< LOD** | **< LOD** | **< LOD** | **< LOD** | **-0.24** | **-1.29** | **-39.77** |
| 31 | 1.004 | 129 | 244 | 2 | 10 | 1 | **< LOD** | 20 | 162 | 4 | 4.47 | 4.03 | -25.70 |
| 32 | 1.030 | 2185 | 1262 | 7 | 15 | 6 | 57 | 21745 | 819 | 27 | 4.29 | 1.59 | -24.20 |
| 33 | 1.024 | 1510 | 1469 | 23 | 77 | 2 | 1 | 140 | 338 | 40 | 4.99 | 2.43 | -24.43 |
| 34 | 1.018 | 892 | 610 | 30 | 67 | 6 | 5 | 104 | 717 | 12 | 4.98 | 4.06 | -25.64 |
| 35 | 1.012 | 787 | 756 | 6 | 10 | 3 | 5 | 120 | 656 | 13 | 4.53 | 4.21 | -25.12 |
| 36 | 1.007 | 544 | 292 | 7 | 8 | 1 | 2 | 299 | 256 | 9 | 4.71 | 3.79 | -25.86 |
| 37 | 1.004 | 268 | 255 | 8 | 24 | 8 | 5 | 38 | 185 | **< LOD** | 5.38 | ND | -24.91 |
| **38** | **1.010** | **< LOD** | **< LOD** | **< LOD** | **< LOD** | **< LOD** | **< LOD** | **< LOD** | **< LOD** | **< LOD** | **0.13** | **ND** | **-29.73** |
| 39 | 1.005 | 340 | 298 | 9 | 38 | 5 | 11 | 45 | 231 | 5 | 3.71 | 3.04 | -25.25 |
| 40 | 1.002 | 85 | 123 | 1 | 2 | 1 | 1 | 92 | 51 | **< LOD** | 4.26 | 3.87 | -26.05 |
| 41 | 1.018 | 613 | 674 | 4 | 12 | 3 | 2 | 168 | 260 | 16 | 4.83 | 3.24 | -24.03 |
| 42 | 1.012 | 664 | 1075 | 9 | 13 | 2 | 1 | 81 | 349 | 13 | 5.07 | 4.49 | -24.32 |
| 43 | 1.014 | 2302 | 1184 | 46 | 92 | 35 | 37 | 337 | 1662 | 37 | 4.49 | 3.20 | -24.76 |
| 44 | 1.006 | 763 | 546 | 12 | 9 | 1 | 10 | 45 | 227 | 7 | 3.65 | 2.69 | -25.38 |
| 45 | 1.022 | 3192 | 3260 | 65 | 366 | 39 | 34 | 642 | 1265 | 81 | 4.42 | 2.19 | -25.01 |
| 46 | 1.026 | 4059 | 1958 | 90 | 202 | 64 | 32 | 341 | 1153 | 63 | 4.29 | 2.08 | -24.30 |
| **47** | **1.014** | **< LOD** | **< LOD** | **< LOD** | **< LOD** | **< LOD** | **< LOD** | **< LOD** | **< LOD** | **< LOD** | **0.00** | **-1.10** | **-39.07** |
| 48 | 1.009 | 133 | 313 | 1 | 16 | 1 | **< LOD** | 73 | 98 | 3 | 3.87 | 4.41 | -25.29 |
| 49 | 1.013 | 699 | 728 | 10 | 12 | 4 | 11 | 1304 | 645 | 13 | 4.84 | 3.57 | -24.34 |
| 50 | 1.007 | 645 | 799 | 8 | 33 | 3 | 2 | 201 | 322 | 13 | 4.60 | 4.06 | -24.57 |
| **51** | **1.013** | **< LOD** | **< LOD** | **< LOD** | **< LOD** | **< LOD** | **< LOD** | **< LOD** | **< LOD** | **< LOD** | **-0.43** | **-2.67** | **-38.66** |

LOD (GC-MS/MS): A, Etio, PD, 11-OH-A: all 5 ng/ml; 5α and 5βAdiol: both 1 ng/ml; T and E; both 0.5 ng/ml; DHEA: 2,5 ng/ml. ND = no detectable amount (dixanthylurea-precipitate) for EA-IRMS.
