# Supplementary material for: Identification of Synthetic Urine by Analysis of Stable Carbon and Nitrogen Isotope Ratios and Comparison to Established GC‐MS/MS and LC‐MS/MS Analysis
Source: Drug Test Anal. 2026 Jun 18;18(8):1145–51. doi: 10.1002/dta.70106 (PMC13432729; doi:10.1002/dta.70106)
Supplement: Supplementary file 3 — Table S3: Specific gravity (SG), concentrations of endogenous steroids (androsterone [A], etiocholanolone [Etio], 5α‐androstane‐3α,17β‐diol [5αAdiol], 5β‐androstane‐3α,17β‐diol [5βAdiol], testosterone [T], epitestosterone [E], pregnanediol [PD], 11β‐hydroxy‐androsterone [11‐OH‐A], and dehydroepiandrosterone [DHEA]) and δ15N and δ13C results for specimen set B. SU = synthetic urine; AU = authentic urine, SU:AU = percentage of mixtures. [file DTA-18-1145-s004.docx]

Table S3: Specific gravity (SG), concentrations of endogenous steroids (androsterone (A), etiocholanolone (Etio), 5α-androstane-3α,17β-diol (5αAdiol), 5β-androstane-3α,17β-diol (5βAdiol), testosterone (T), epitestosterone (E), pregnanediol (PD), 11β-hydroxy-androsterone (11-OH-A) and dehydroepiandrosterone (DHEA)) and δ^15^N and δ^13^C results for specimen set B. SU = synthetic urine; AU = authentic urine, SU:AU = percentage of mixtures.

| **Sample** | **Product** | **SU:AU** | **SG** | **A** | **Etio** | **5αAdiol** | **5βAdiol** | **T** | **E** | **PD** | **11-OH-A** | **DHEA** | **δ^15^N_total_** | **δ^15^N_urea_** | **δ^13^C_total_** |
| --- | --- | --- | --- | --- | --- | --- | --- | --- | --- | --- | --- | --- | --- | --- | --- |
|  |  |  |  | **ng/mL** | | | | | | | | | **‰** | | |
| 52 | 1 | 90:10 | 1.015 | 107 | 101 | 2 | 7 | 1 | 1 | 156 | 73 | **< LOD** | 0.28 | -1.04 | -37.52 |
| 53 |  | 50:50 | 1.014 | 616 | 573 | 11 | 35 | 4 | 6 | 997 | 371 | 10 | 2.04 | 1.23 | -30.43 |
| 54 |  | 10:90 | 1.014 | 1163 | 1051 | 21 | 64 | 8 | 10 | 1819 | 633 | 21 | 4.18 | 3.56 | -25.84 |
| 55 | 2 | 90:10 | 1.013 | 106 | 100 | 2 | 7 | 1 | 1 | 153 | 71 | **< LOD** | 0.20 | -0.78 | -37.32 |
| 56 |  | 50:50 | 1.013 | 619 | 568 | 11 | 36 | 5 | 5 | 955 | 371 | 10 | 2.25 | 1.44 | -30.13 |
| 57 |  | 10:90 | 1.013 | 1142 | 1029 | 21 | 65 | 8 | 10 | 1798 | 652 | 20 | 4.27 | 3.61 | -25.45 |
| 58 | 3 | 90:10 | 1.014 | 105 | 95 | 2 | 7 | 1 | 1 | 156 | 69 | **< LOD** | 0.21 | -0.79 | -36.87 |
| 59 |  | 50:50 | 1.014 | 606 | 565 | 11 | 36 | 5 | 6 | 975 | 354 | 10 | 2.16 | 1.39 | -29.38 |
| 60 |  | 10:90 | 1.014 | 1143 | 1034 | 21 | 67 | 8 | 10 | 1770 | 642 | 20 | 4.01 | 3.63 | -25.78 |
| 61 | 4 | 90:10 | 1.019 | 109 | 101 | 2 | 7 | 1 | 1 | 152 | 75 | **< LOD** | 0.43 | -2.29 | -35.52 |
| 62 |  | 50:50 | 1.016 | 623 | 578 | 11 | 35 | 4 | 5 | 957 | 363 | 10 | 1.80 | -0.58 | -30.63 |
| 63 |  | 10:90 | 1.014 | 1246 | 1136 | 23 | 73 | 8 | 11 | 1860 | 695 | 24 | 3.91 | 3.05 | -25.72 |
| 64 | 5 | 90:10 | 1.013 | 110 | 101 | 2 | 7 | 1 | 1 | 147 | 70 | **< LOD** | 0.18 | -2.13 | -37.19 |
| 65 |  | 50:50 | 1.013 | 652 | 600 | 11 | 36 | 5 | 6 | 973 | 370 | 10 | 1.65 | -0.01 | -31.05 |
| 66 |  | 10:90 | 1.014 | 1186 | 1073 | 20 | 65 | 8 | 10 | 1791 | 679 | 20 | 3.97 | 3.33 | -25.74 |
| 67 | 6 | 90:10 | 1.010 | 112 | 103 | 2 | 7 | 1 | 1 | 142 | 59 | **< LOD** | 2.70 | 3.54 | -24.91 |
| 68 |  | 50:50 | 1.012 | 613 | 570 | 11 | 34 | 4 | 5 | 934 | 371 | 10 | 4.38 | 4.34 | -24.60 |
| 69 |  | 10:90 | 1.013 | 1160 | 1053 | 21 | 66 | 8 | 10 | 1778 | 661 | 21 | 4.80 | 4.25 | -24.66 |
| 70 | 7 | 90:10 | 1.015 | 109 | 102 | 2 | 7 | 1 | 1 | 152 | 72 | **< LOD** | 0.29 | -0.19 | -32.54 |
| 71 |  | 50:50 | 1.014 | 628 | 584 | 11 | 35 | 4 | 5 | 964 | 371 | 11 | 2.22 | 1.25 | -28.26 |
| 72 |  | 10:90 | 1.014 | 1133 | 1020 | 20 | 62 | 7 | 10 | 1729 | 625 | 21 | 4.09 | 3.62 | -25.23 |
| 73 | 8 | 90:10 | 1.015 | 109 | 101 | 2 | 7 | 1 | 1 | 151 | 69 | **< LOD** | 0.28 | -0.64 | -32.84 |
| 74 |  | 50:50 | 1.014 | 628 | 576 | 12 | 37 | 4 | 5 | 954 | 386 | 11 | 2.00 | 1.41 | -28.89 |
| 75 |  | 10:90 | 1.014 | 1121 | 1025 | 22 | 66 | 8 | 10 | 1771 | 630 | 20 | 4.13 | 3.60 | -25.34 |

LOD (GC-MS/MS): A, Etio, PD, 11-OH-A: all 5 ng/ml; 5α and 5βAdiol: both 1 ng/ml; T and E; both 0.5 ng/ml; DHEA: 2,5 ng/ml.
