# Supplementary material for: Identification of Synthetic Urine by Analysis of Stable Carbon and Nitrogen Isotope Ratios and Comparison to Established GC‐MS/MS and LC‐MS/MS Analysis
Source: Drug Test Anal. 2026 Jun 18;18(8):1145–51. doi: 10.1002/dta.70106 (PMC13432729; doi:10.1002/dta.70106)
Supplement: Supplementary file 4 — Table S4: Evaluation of specimen set B by LC‐MS/MS. Specimen set B consisted of mixtures of synthetic urine (SU) and authentic urine (AU) in the rations 90:10, 50:50, and 10:90. Given are measured creatinine values (CREA), number of endogenous biomolecule (EB) detections, and detected direct SU markers. [file DTA-18-1145-s002.docx]

Table S4: Evaluation of specimen set B by LC-MS/MS. Specimen set B consisted of mixtures of synthetic urine (SU) and authentic urine (AU) in the rations 90:10, 50:50 and 10:90. Given are measured creatinine values (CREA), number of endogenous biomolecule (EB) detections and detected direct SU markers.

| **Sample** | **Product** | **SU:AU** | **CREA** | **LC-MS/MS** | | |
| --- | --- | --- | --- | --- | --- | --- |
|  |  |  | **mg/L** | **EB** | **SU Marker** | **Evaluation** |
| 52 | 1 | 90:10 | 470 | 4 | SUM255 | Not AU, diluted with SU |
| 53 |  | 50:50 | 670 | 8 | (SUM255) | AU, potentially diluted with SU |
| 54 |  | 10:90 | 830 | 8 | No | AU |
| 55 | 2 | 90:10 | 360 | 3 | SUM255 | Not AU, diluted with SU |
| 56 |  | 50:50 | 640 | 8 | (SUM255) | AU, potentially diluted with SU |
| 57 |  | 10:90 | 810 | 9 | No | AU |
| 58 | 3 | 90:10 | 420 | 3 | (SUM255) | Not AU, potentially diluted with SU |
| 59 |  | 50:50 | 670 | 9 | No | AU |
| 60 |  | 10:90 | 870 | 9 | No | AU |
| 61 | 4 | 90:10 | 1120 | 3 | No* | Not AU |
| 62 |  | 50:50 | 1040 | 8 | No* | AU |
| 63 |  | 10:90 | 890 | 9 | No* | AU |
| 64 | 5 | 90:10 | 930 | 3 | No* | Not AU |
| 65 |  | 50:50 | 910 | 7 | No* | AU |
| 66 |  | 10:90 | 850 | 7 | No* | AU |
| 67 | 6 | 90:10 | 90 | 4 | No* | Not AU |
| 68 |  | 50:50 | 450 | 8 | No* | AU |
| 69 |  | 10:90 | 780 | 8 | No* | AU |
| 70 | 7 | 90:10 | 560 | 3 | PPG | Not AU, diluted with SU |
| 71 |  | 50:50 | 700 | 6 | PPG | AU diluted with SU |
| 72 |  | 10:90 | 820 | 8 | (PPG) | AU, potentially diluted with SU |
| 73 | 8 | 90:10 | 650 | 3 | PPG | Not AU, diluted with SU |
| 74 |  | 50:50 | 750 | 7 | PPG | AU diluted with SU |
| 75 |  | 10:90 | 840 | 8 | (PPG) | AU, potentially diluted with SU |

*no known direct synthetic urine markers for SU products SU4, SU5, SU6
